# Innovations in thoracic imaging: CT, radiomics, AI and x‐ray velocimetry

**DOI:** 10.1111/resp.14344

**Published:** 2022-08-14

**Authors:** Rozemarijn Vliegenthart, Andreas Fouras, Colin Jacobs, Nickolas Papanikolaou

**Affiliations:** ^1^ Department of Radiology University of Groningen, University Medical Center Groningen Groningen the Netherlands; ^2^ Data Science in Health (DASH) University of Groningen, University Medical Center Groningen Groningen the Netherlands; ^3^ 4DMedical Los Angeles California USA; ^4^ Department of Medical Imaging Radboud University Medical Center Nijmegen the Netherlands; ^5^ Champalimaud Research Champalimaud Foundation Lisbon Portugal; ^6^ AI Hub The Royal Marsden NHS Foundation Trust London UK; ^7^ The Institute of Cancer Research London UK

**Keywords:** computed tomography, deep learning, lung cancer, lung nodules, machine learning, radiomics, x‐ray velocimetry

## Abstract

In recent years, pulmonary imaging has seen enormous progress, with the introduction, validation and implementation of new hardware and software. There is a general trend from mere visual evaluation of radiological images to quantification of abnormalities and biomarkers, and assessment of ‘non visual’ markers that contribute to establishing diagnosis or prognosis. Important catalysts to these developments in thoracic imaging include new indications (like computed tomography [CT] lung cancer screening) and the COVID‐19 pandemic. This review focuses on developments in CT, radiomics, artificial intelligence (AI) and x‐ray velocimetry for imaging of the lungs. Recent developments in CT include the potential for ultra‐low‐dose CT imaging for lung nodules, and the advent of a new generation of CT systems based on photon‐counting detector technology. Radiomics has demonstrated potential towards predictive and prognostic tasks particularly in lung cancer, previously not achievable by visual inspection by radiologists, exploiting high dimensional patterns (mostly texture related) on medical imaging data. Deep learning technology has revolutionized the field of AI and as a result, performance of AI algorithms is approaching human performance for an increasing number of specific tasks. X‐ray velocimetry integrates x‐ray (fluoroscopic) imaging with unique image processing to produce quantitative four dimensional measurement of lung tissue motion, and accurate calculations of lung ventilation.

## Abstract 2

**See related** Editorial

**Abbreviations**
AI: artificial intelligence
ARDS: Acute respiratory distress syndrome
CF: cystic fibrosis
CT: computed tomography
CXR: chest radiography
EGFR: epidermal growth factor receptor
FEV_1_
: forced expiratory volume in 1 s
FVC: forced vital capacity
kVp: tube voltage peak
mAs: milliampere‐seconds
NSCLC: non‐small cell lung cancer
PACS: picture archiving and communications system
PCD: photon‐counting detector
ULD: ultra‐low‐dose
XV: x‐ray velocimetry
WHO: World Health Organization

## INTRODUCTION

In recent years, pulmonary imaging has seen enormous progress, with the introduction, validation and implementation of new hardware and software, some of which are discussed in this review. There is a general trend from mere visual evaluation of radiological images to quantification of abnormalities and biomarkers, and assessment of ‘non visual’ markers that contribute to establishing diagnosis or prognosis. Important catalysts to the developments in thoracic imaging include new indications (like computed tomography [CT] lung cancer screening)^1^, ^2^ and the COVID‐19 pandemic^3^; both have led to large increases in numbers of chest examinations in the (very) recent past.

In the Respirology Invited Review Series on Thoracic Imaging, this article complements the recent review on functional MRI,^4^ and describes developments in CT, radiomics, artificial intelligence (AI) and x‐ray velocimetry, with particular focus on lung nodules/tumours (CT, radiomics and AI) and ventilation (x‐ray velocimetry).

## COMPUTED TOMOGRAPHY

In recent years, technical improvements in CT, including developments in—among others—detector technology, temporal resolution, image reconstruction techniques and spectral imaging methods, have led to a surge in validation, accuracy and implementation studies. In this review, the focus is on ultra‐low‐dose (ULD) imaging and photon‐counting CT scanning. A third important topic of progress is so‐called spectral or dual‐energy CT. This technique, its status, and its current applications have been extensively reviewed in a recent overview article, to which readers are kindly referred.^5^ Spectral CT is used in pulmonary embolism suspicion for improved image quality and assessment of pulmonary perfusion, and can yield additional information in pulmonary hypertension. Furthermore, blood volume information from spectral CT can help to distinguish parenchymal pathologies like pneumonia or infarct. In lung cancer, spectral CT can be used for tumour perfusion evaluation, and help to assess treatment response.

### ULD imaging

With recent improvements in CT hardware and software, there has been increasing interest in so‐called ULD imaging. This term is generally considered to apply to CT scans with a calculated radiation dose of below 1 milliSievert.^1^ ‘As low as reasonably achievable’ is a central radiological principle, because of the known association between radiation dose and cancer incidence. However, there is a trade‐off between how much the radiation dose can be lowered, and the resulting image quality. The lower the dose, the noisier the CT images, where small details with low contrast may become missed, and strain for radiologists to read the scan increases. The image quality should remain sufficient to allow accurate evaluation of the primary indication of the CT scan.^6^

The lung is a high contrast organ, which makes it appropriate for ULD CT, in particular for the evaluation of solid lung nodule/pathology against the air‐filled lungs. There is particular interest in ULD imaging due to the recent recommendations to prepare and/or implement CT lung cancer screening in high‐risk individuals.^1^, ^2^ Importantly, this screening takes place in an apparently healthy population, and thus, concerns need to be addressed regarding potential risks of radiation dose due to the repeated CT screenings.^7^

#### Solutions to lower the radiation dose

In order to reduce the radiation dose in chest CT to an ULD setting, different solutions can be applied, depending on the specific CT vendor. See the overview in Box 1, with explanation of radiological terms. Reducing radiation dose starts by carefully considering the indication of the CT scan, selection of the appropriate scan protocol and limiting the scan range to the area in question. The basic CT scan acquisition parameters include the tube voltage peak (kVp) and the tube current time product (milliampere‐seconds, mAs). Reducing these parameters result in lower‐energy x‐ray photons with less penetrative power and less photon output, in particular less low energy photons; this reduces radiation dose. Another method to decrease photons that have limited contribution to image information is the use of a thin tin filter between the x‐ray tube and the scanned individual.

BOX 1 **Solutions resulting in or allowing radiation dose reduction in routine chest CT**

**Table:** 

Parameter	Description of parameter	Intervention
*CT scanner related*		
Tube current time product, milliampere‐seconds (mAs)	A measure of the quantity of x‐ray photons produced per second	Reduce mAs use tube‐current modulation
Tube voltage peak (kVp)	Determines maximum and average energy of x‐ray photons, and photon quantity	Reduce kVp patient specific kVp selection
Tin filtration	Metal sheet pre‐patient that removes low‐energy photons	Apply tin filter
*Image reconstruction related*		
Iterative reconstruction (IR)	Specific methods of image reconstruction, where an initial guess of the CT data is adjusted in several iterations in order to match the measured CT data until the difference is smaller than a preset value; usually these methods are provided in different user selectable strengths	Apply (a level of) IR
Deep learning reconstruction (DLR)	Image reconstruction methods, some vendor‐specific and some stand‐alone, based on deep learning training networks, that transform noisy, (ultra‐)low‐dose images into high‐quality images	Apply (a level of) DLR

All described techniques, however, also result in less photons reaching the detector, with increased noise and less information in the eventual image. This is only partly neutralized by improved detector systems. Without other interventions, (very) low radiation dose leads to high noise in the image, limiting the diagnostic accuracy. In particular the combination with new image reconstruction techniques, so‐called iterative reconstruction techniques,^8^ and more recently, deep‐learning based techniques,^9^ can counteract the image noise and reduced image information.

**FIGURE 1:**
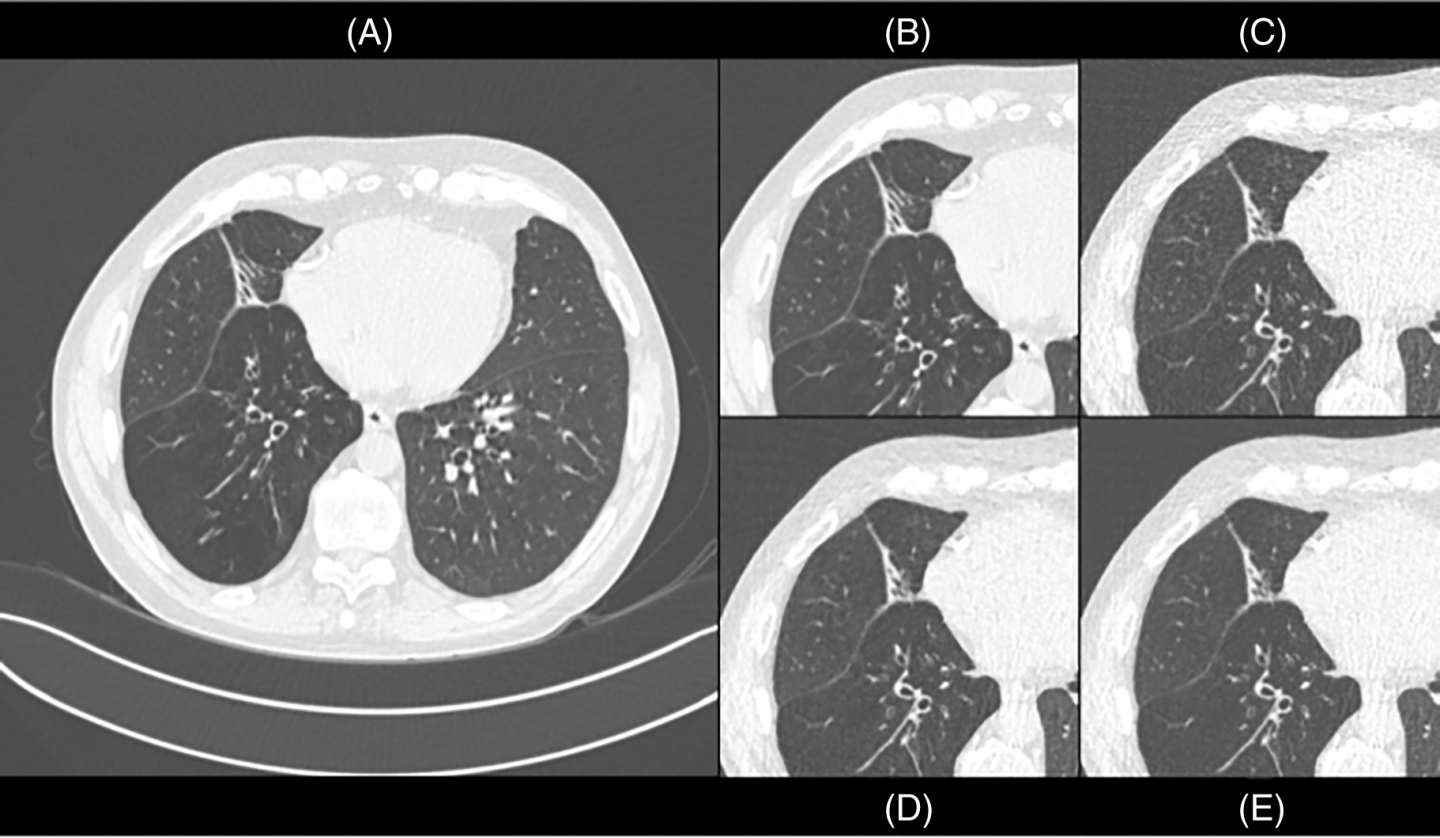
Standard dose CT (HRCT) and ultra‐low‐dose CT (ULD) in the same patient (from the cohort described in Reference 10). (A) Shows a standard reconstructed HRCT image (filtered back projection). (B) Shows a cropped view of the standard reconstructed HRCT image. (C) Shows a standard reconstructed ULD CT image, with elevated image noise. (D) Shows an ULD CT image based on deep learning reconstruction. (E) Shows an ULD CT image based on iterative reconstruction. (D) and (E) show less image noise, more similar to standard dose (HRCT) image

In iterative reconstruction, an initial guess of the CT data is adjusted in several iterations in order to match the measured CT data until the difference is smaller than a preset value. Usually these methods are provided in different user selectable strengths. By now, there are different generations of iterative reconstructions. Furthermore, there are now deep learning training networks that transform noisy, (ultra‐)low‐dose images into high‐quality images. For an example, see Figure 1.

Studies on ULD in chest CT so far have mainly focused on the image quality and the accuracy for detection of lung nodules.^11^, ^12^, ^13^, ^14^, ^15^, ^16^, ^17^ The most recent studies have applied combinations of techniques described above, in particular, tin filtration with newest generations of iterative reconstruction,^11^, ^12^, ^13^ or reduced tube current with deep‐learning reconstruction.^14^, ^15^ In these studies 40–200 patients underwent a clinically indicated, regular dose chest CT scan and in the same session an ULD CT scan. The calculated radiation dose of the ULD CT scan was generally 0.15–0.2 mSv, which is around 90% radiation dose reduction compared to a routine non‐contrast chest CT, and getting close to the radiation dose of a chest x‐ray examination (0.05–0.1 mSv); this is a remarkable feat.

Overall, the sensitivity of detection of solid lung nodules in ULD CT was similar or slightly lower (10%–17% reduced) compared to regular dose CT.^11^, ^12^, ^13^, ^14^ It is important to realize that for sensitive detection of subsolid nodules, the lowest radiation dose in ULD CT does not suffice, as conspicuity of these lesions becomes very limited.^14^, ^18^, ^19^ Further research is needed on what combination of scan protocol specifications (in tube voltage, tube current and reconstruction, with or without x‐ray beam filtration) is needed to obtain a sufficiently high level of nodule detectability. Likely, computer automated detection of lung nodules, whether or not AI‐based, may help in this respect.^11^, ^13^

There are conflicting results and insufficient evidence on the effect of BMI on the accuracy of lung nodule detection. Some studies indicate no negative effect for individuals with a BMI above 25,^11^, ^20^ while another recent study showed significantly lower detectability of nodules at higher BMI.^14^ Some other studies excluded individuals with high BMI.^16^, ^17^

An important point that so far has received limited attention is the potential effect of ULD CT acquisition on quantification of lung nodules (volume, diameter and density). These issues need further research and optimization prior to potential implementation in screening settings, to make sure that lung cancer screening outcomes are not affected.

#### ULD chest CT for other applications than lung nodules

ULD CT protocols could potentially also be used for indications other than evaluation/follow‐up of lung nodules. Regarding detection and quantification of emphysema, two studies which included a more general chest CT patient cohort^21^ and a COPD cohort^10^ showed that ULD CT combined with iterative reconstruction (IR) or deep learning reconstruction (DLR) can indeed detect lesser and more severe emphysema, without significant difference in sensitivity to standard dose CT. However, some differences in the extent of emphysema resulted, depending on the reconstruction level. Thus, consistent scan protocols are needed for follow‐up. Furthermore, higher BMI may result in lower detectability of emphysema.^20^ Interestingly, an ULD CT protocol may also be accurate for evaluation of cystic fibrosis (CF), follow up imaging and detection of viral pneumonia, including COVID‐19 pneumonia.^6^, ^22^ However, if the primary indication is interstitial lung disease/abnormality, then an ULD CT protocol is not recommended.^6^

### Photon‐counting detector CT scanner

While innovations in CT hardware and software have been extensive in the last decade, even the newest clinical CT generations have some limitations. Suboptimal spatial resolution hampers evaluation of small structures such as the pulmonary interstitium. In certain patients, the CT scan can show reduced image contrast, significant noise or streak artefacts.^23^ Dual‐energy CT has gained momentum for material differentiation and for functional information but each CT vendor solution has inherent limitations.

Photon‐counting detector (PCD) CT is a ground‐breaking development that will contribute to the solution of these main limitations in CT. In regular CT scanners, x‐ray photons are absorbed in a scintillator detector that converts x‐rays into light photons. Light is then absorbed by an underlying photodiode, and an electrical charge generated, which results in an image signal. In PCD CT, x‐ray photons are absorbed in a semiconductor material. There, positive and negative charges are created that are pulled apart in a strong electric field, instead of an intermediate step of light photons. In contrast to detectors in regular CT scanners, each individual x‐ray photon with its energy is counted. Furthermore, the PCD has a higher geometric dose efficiency, for example by electronic noise suppression.

The technological advances in PCD CT result in higher contrast‐to‐noise ratio, higher spatial resolution (currently down to 0.28 × 0.28 mm in clinical settings), lower radiation dose, reduction of noise and artefacts and improved dual‐energy imaging, with the potential of multi‐energy imaging.^23^ In a recent phantom study on lung nodules, PCD CT yielded higher subjective and objective image quality compared to a regular CT scanner at the same low radiation dose.^24^ First clinical examples in chest CT, as reviewed by Si‐Mohamed,^25^ show excellent spatial resolution of small parenchymal structures, and improved detection of low density structures like subsolid nodules. The first patient studies on the advantages of clinical PCD CT scanners in chest imaging are eagerly awaited.

## RADIOMICS

### Radiomics pipeline

Recently, texture analysis techniques have attracted interest to address a well‐known problem with visual inspection of medical images, namely subjective evaluation and diagnosis.^26^, ^27^ The new paradigm, called radiomics,^28^, ^29^ is based on the transformation of medical images into minable data that became feasible by applying mathematical transformations on specific areas of tissue, primarily tumoral.

**FIGURE 2:**
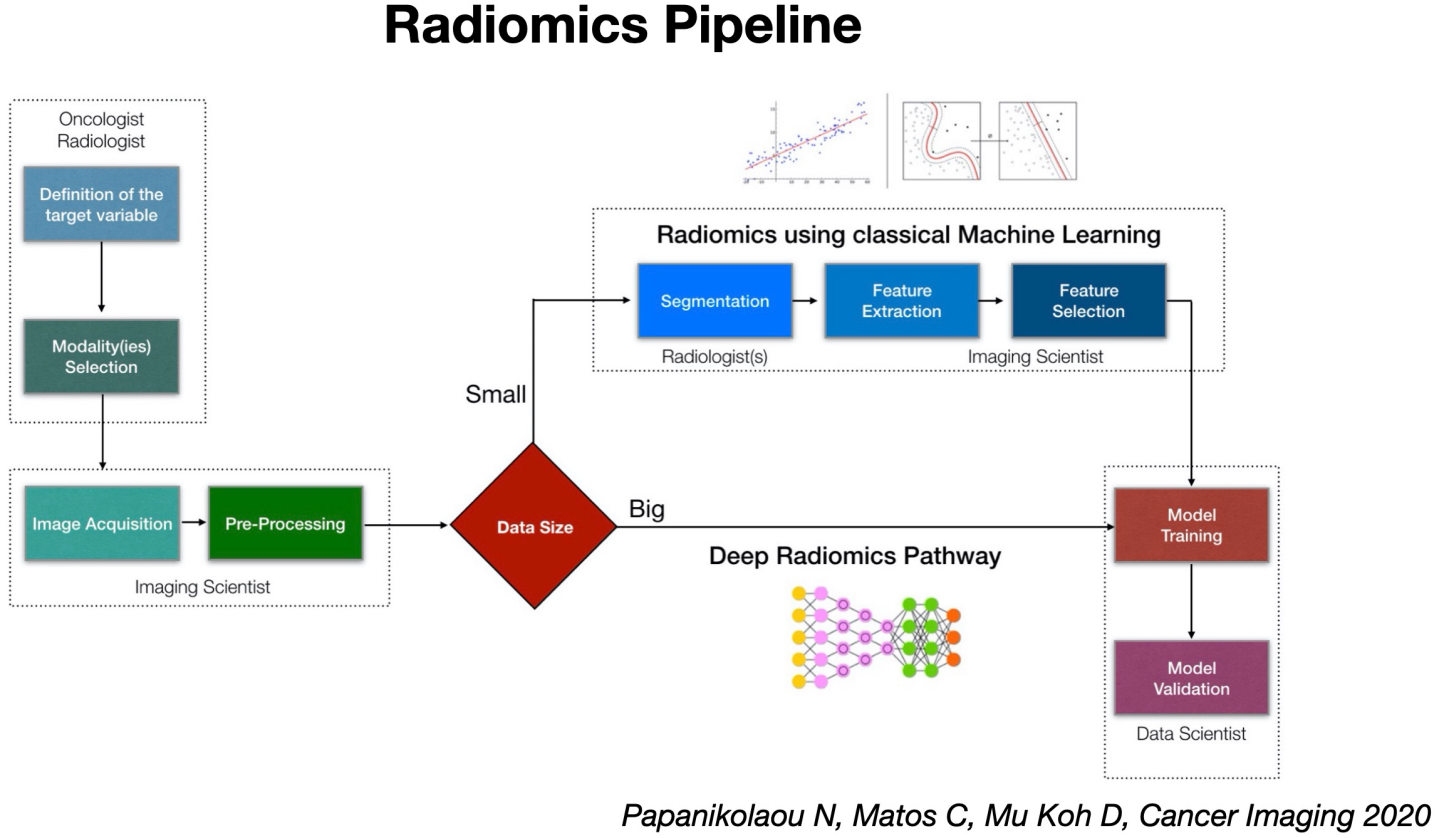
Consecutive steps in a typical radiomics workflow (adapted from reference 30)

The radiomics workflow is comprised of several consecutive phases (Figure 2).^30^ First, a medical oncologist needs to provide a well‐described clinically meaningful case, that addresses an unmet clinical problem related to the patient with (lung) cancer. Consequently, the radiologist needs to identify the relevant data source, namely the type of modality to be considered for data extraction, as well as the type of individual imaging techniques that are relevant to the clinical question under investigation. Next, an imaging scientist will verify the state of the raw data and whether they need any kind of pre‐processing actions before feature extraction. Regarding the data, three important requirements need to be fulfilled: adequate quantity, expected quality and large diversity. As far as data quantity is concerned, it is not possible to provide some hard numbers, however the more data we have, the more efficient the learning process will be. In addition, large datasets can support the training of robust models that are stable to changes and less sensitive to overfitting.

One potential bottleneck in the radiomics workflow is the necessity to provide segmentation masks, preferably of the entire tumour and sometimes even in its vicinity (penumbra). Since tumour segmentation is based on the subjective opinion of radiologists on the exact borders of the tumour, significant variability between readers can be found. To address the latter, it is advised to collaborate with two or more radiologists to provide segmentation masks.

Manual tumour segmentation can be time consuming, and it certainly should be done by expert radiologists which poses issues of availability. The latter problem has initiated a significant effort towards developing automatic or semi‐automatic tools to reduce the work burden and accelerate the segmentation process. These tools are mostly based on deep learning algorithms, where with a moderate number of cases such networks can be trained to produce segmentation masks that then only need to be verified and validated by expert radiologists.

Following the delineation of the tumour, a radiomic feature library or package can be used to extract well defined features that are based on known mathematical formulas. These features can be summarized into three distinct types: first order features, shape features and texture features. First order features estimate properties of individual voxel values, ignoring the spatial interaction between neighbouring voxels, so they are very similar to histogram metrics. Furthermore, there is a group of features that convey two‐ and three‐dimensional shape and volume information. Those can be also used to study purely morphological characteristics of the tumour in an objective manner.

Finally, a vast amount of radiomic features are extracted by applying texture matrices in the images providing information about local and global heterogeneity of the tumour. For example, the grey level co‐occurrence matrix describes the second‐order joint probability function of an image region constrained by the mask. Each element of this matrix represents the number of times the combination of predefined pair of intensities occurs in two pixels in the image that are separated by a distance of 𝛿 pixels along angle 𝜃.

#### Feature selection

In radiomics, as in many other multi‐dimensional problems, usually the number of predictors significantly outweighs the number of samples (*p* ≫ *n*). There is a need therefore to reduce the dimensionality of the problem by identifying the most informative features through a methodology that is called feature selection.

Feature selection is generally performed in consecutive phases. For example, one can use spatial and/or temporal stability analysis as a first step towards dimensionality reduction. Since segmentation masks may differ between radiologists, often at least two segmentation masks are obtained from the same lesion. Then, the computed radiomic features are analysed with interobserver correlation coefficients to identify those above a certain threshold (usually 0.75) meaning that their value is not significantly changing across the two radiologists. After stability analysis, a zero or near zero variance method can be used to remove features that are constant across exams of different patients. Subsequently, correlation analysis may detect redundant features and remove them. Finally, more sophisticated methods like minimum redundancy maximum relevance or recursive feature elimination are used to craft the radiomic signature.^30^

#### Model selection/assessment

According to the no free lunch theorem in machine learning, ‘no single machine learning algorithm works best for every problem’.^31^ This means that we do not know in advance which algorithm is the most efficient for each individual problem. Of course, someone may have a strategy to choose which type of algorithms to focus on, for example if performance at the expense of ‘explainability’ is the target, to consider deep learning instead of conventional machine learning algorithms.

In any case, we need to train several algorithms and compare them to make a model selection. This is a very tricky task, since often someone might use the wrong type of dataset to make the model selection, thereby increasing bias and resulting in models with poor generalizability. The selection of the best algorithm is based on the internal validation process, using part of the data to measure performance. In addition and depending on the problem, the interpretability and transparency of each algorithm may play a role on the final selection.

One of the main obstacles in radiomics is the low generalizability, not only due to incorrect practices, but also due to the vast variability of the feature representations that might reflect differences between scanner vendors, scanner models and even acquisition protocols. Contrary to other domains in healthcare, radiomics is suffering from this lack of standardized inputs to machine learning models; this may be responsible for the low clinical usability. It is not a surprise that although thousands of radiomic models have been published, only a very small minority might end up useful in clinical practice.

### Clinical applications of radiomics/radiogenomics in lung cancer

Lung cancer was among the first fields at which radiomics was aimed.^32^, ^33^ The most common modalities were CT and Positron‐Emission Tomography‐CT, given their leading role in the clinical diagnostic workup of patients with lung cancer.

In 2014, Aerts et al. were the first to develop a radiomic signature to predict overall survival in patients with lung cancer.^34^ They used data from 422 patients with non‐small cell lung cancer (NSCLC) from Maastro Clinic to construct the radiomic signature, which they validated using another dataset from Radboud University comprising 225 patients. In addition, they successfully validated their signature based on two other datasets comprising patients with head and neck cancer.

An important part of this work was the biological validation that was done assessing associations and correlations between radiomic features and gene expressions. In 2018, a publication by another group challenged this radiomic signature by arguing that the presented radiomic signature was predictive of the tumour volume and not overall survival.^35^ Furthermore, radiomics was shown to be able to quantify tumour phenotypic characteristics non‐invasively. In an early study by Wu et al. published in 2016,^33^ they investigated the association between radiomic features and the tumour histologic subtypes (adenocarcinoma and squamous cell carcinoma). To predict histologic subtypes, they employed various machine‐learning algorithms and feature selection techniques while they independently evaluated prediction performance.

More recently several studies associating or combining radiomics and genomics, termed as radiogenomics, have emerged. Zhou et al.^36^ showed that CT Hounsfield attenuation measurements and lesion margins were correlated with cell‐cycle genes, while the presence of irregular borders and ground glass opacities in the lesion was correlated with epidermal growth factor receptor (EGFR) expression. In a study by Rizzo et al.,^37^ CT features such as the presence of air bronchogram, pleural retraction, small lesion size and absence of fibrosis were associated with EGFR mutation, whereas pleural effusion was associated with anaplastic lymphoma kinase mutation. Round shape, nodules in non‐tumour lobes and smoking were variables linked to KRAS mutation.

Other, less frequent mutations such as RET and ROS1, which comprise 1%–2% of all lung adenocarcinomas, have also been assessed for associations with imaging features. In a study of 26 patients, Gevaert et al.^38^ found that the presence of air bronchograms was associated with overexpression of the KRAS oncogene. Weiss et al.^39^ also found that imaging features could predict the KRAS status of patients with NSCLC. As a predictive marker, KRAS has been linked to round shape, nodules in non‐tumour lobes, multiple small nodules, as well as, general radiomic profiles.

## ARTIFICIAL INTELLIGENCE

### The deep learning revolution

AI refers to computer systems that can interpret and/or learn from data to perform certain tasks. Deep learning, a subdomain of machine learning where computers use powerful compute resources to learn high dimensional features directly from large amounts of data, has led to a revolution in the field of AI. Deep learning gained momentum in 2012 when Krizhevsky et al.^40^ showed that a convolutional neural network had beaten (by a substantial margin) the best performing algorithm in the ImageNet Large Scale Visual Recognition Challenge, an annual competition where algorithms compete to correctly classify and detect objects and scenes in natural images.

A crucial difference with previous machine learning methods is that deep learning uses large neural networks with millions of parameters that are learned directly from the raw data and corresponding labels. This is an extremely powerful technology. In many fields, ranging from autonomous driving for the auto industry to medical image interpretation for the healthcare sector, deep learning methods have replaced previous machine learning methods.

#### Deep learning in medical imaging

For medical image interpretation, deep learning has also become the standard methodology of choice for tasks like detection, segmentation and classification.^41^ The performance of deep learning algorithms is reaching or even surpassing human performance for an increasing number of tasks.^41^ Several key exemplary papers have demonstrated this for tasks such as skin lesion classification in dermatology,^42^ diabetic retinopathy detection in ophthalmology,^43^ breast metastasis detection in pathology^44^ and lung cancer detection on low‐dose chest CT screening.^45^, ^46^ The revolution arising from deep learning technology has led to a substantial increase in the number of approved medical devices based on AI in Europe and the United States since 2015, with many being approved for use in radiology.^47^ The sector is still in its infancy; peer‐reviewed evidence on the efficacy of these devices is currently lacking for a large proportion of approved AI products.^48^ With deep learning technology entering the healthcare market, it is important for physicians involved in respiratory care to understand what AI is and how it may affect their field in the near future.

### Current AI applications in thoracic imaging

Several review papers have been published that discuss AI for thoracic imaging,^49^ or for various subfields within thoracic imaging, such as lung cancer screening,^50^ pulmonary nodule management,^51^ chest x‐ray classification,^52^ cardiovascular imaging,^53^ paediatric imaging^54^ or COVID‐19 diagnosis and prognosis from CT and chest radiography (CXR).^55^, ^56^ The application options for AI in thoracic imaging are diverse and extensive. A recent analysis of 100 CE‐approved products in Europe reported that chest radiology is an important area for AI software, with 31 out of 100 products addressing tasks in chest imaging.^48^ We will now cover a few popular applications for AI in thoracic imaging.

#### Publicly available datasets for lung nodules

Deep learning technology needs large amounts of data for training, and therefore, the availability of publicly available datasets is important for development and validation of AI algorithms. When datasets have been released publicly, this also leads to publications from many different research groups, including more fundamental research groups focusing on general computer vision with limited access to medical data.

**FIGURE 3:**
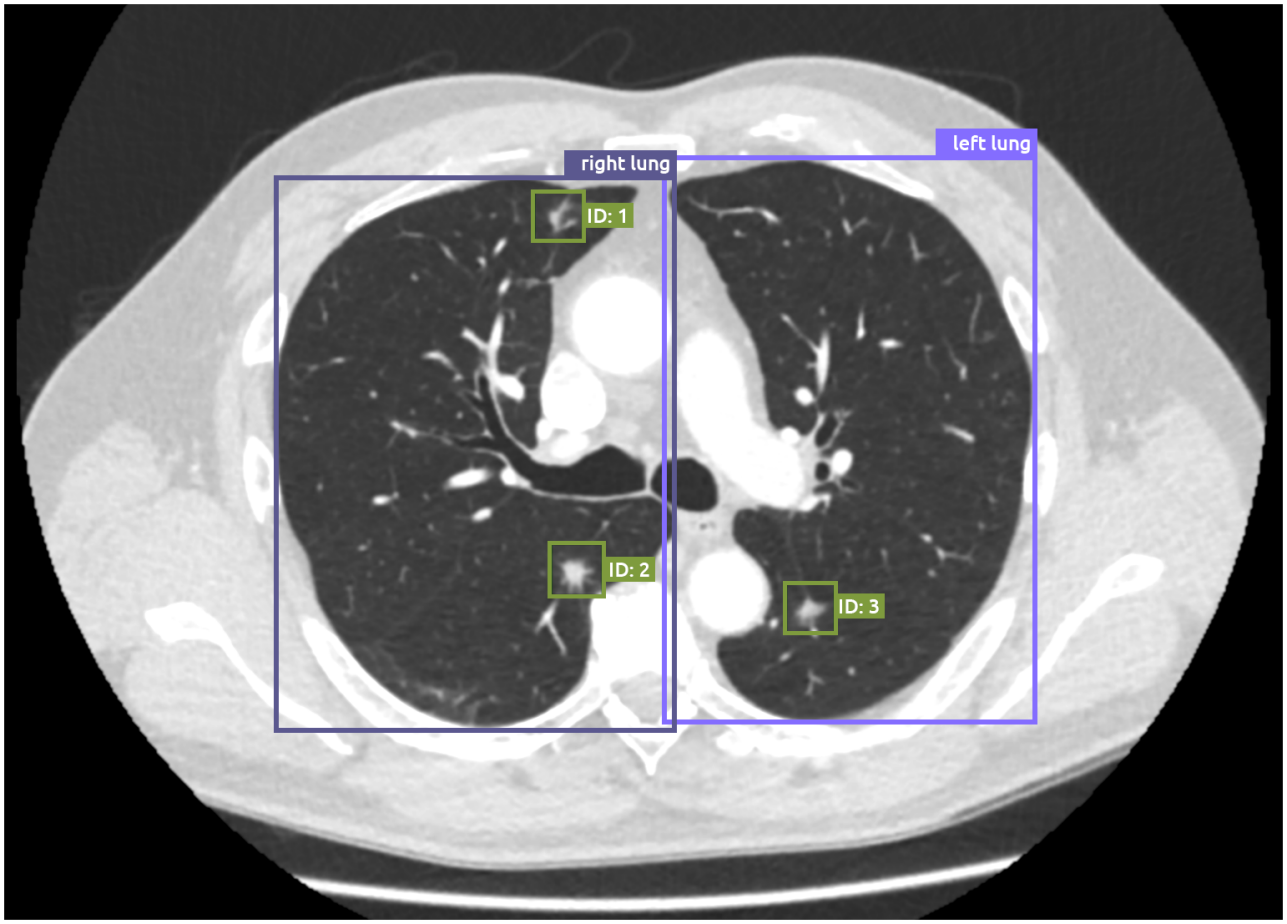
Example of the output of an artificial intelligence (AI) algorithm for lung localization and nodule detection on chest computed tomography (CT) imaging. The lung localization algorithm takes a slice of a CT scan as input, and produces a bounding box around the left lung and the right lung. The lung nodule detection algorithm takes a CT scan as input, and produces bounding boxes around detected lung nodules, which can be presented to radiologists as an aid for the detection of nodules in chest CT images

A classic application that has received a lot of attention is the detection of lung nodules on CXR or CT (Figure 3). The most used publicly available datasets for nodule detection on CT are the Lung Image Database Consortium (LIDC)‐Image Database Resource Initiative (IDRI) database^57^ and the National Lung Screening Trial (NLST) database (also has CXR images).^58^ For CXR, large databases with images have been released, such as the Chest‐Xray14,^59^ CheXpert,^60^ MIMIC‐CXR^61^ and the PadChest^62^ database, each consisting of more than 100 k images. The act of releasing a dataset publicly, preferably in combination with a competition, has been proven as an effective strategy to attract community attention and investigate what approaches work well for a certain application, and what performance can be considered state‐of‐the‐art. Examples of competitions for lung nodules on CT are the LUNA16 challenge,^63^ the SPIE Lung X challenge,^64^ and the Kaggle Data Science Bowl 2017.^46^ The results of the competition are typically described in a scientific publication, which puts the results into context for clinicians and describes what future steps are needed in the field.

#### AI for pneumonia and tuberculosis detection

In addition to the detection of lung nodules, other exemplary applications are the detection of pneumonia and tuberculosis. A pneumonia detection challenge was organized by the Radiology Society of North America, accompanied by the release of 33,000 CXR images with pneumonia annotations, in an attempt to reach out to the machine learning community to develop algorithms that automatically identify potential pneumonia on CXR. The winning solutions are now available as open‐source software and can be taken up and further developed into certified medical products.

Tuberculosis detection is another topic of numerous AI publications. Tuberculosis remains a leading cause of death by infection. To eliminate this disease, improvements in access to tuberculosis diagnostics are needed in developing countries. The World Health Organization (WHO) has identified the use of triage tests as one potential solution for improving tuberculosis diagnostic pathways in resource‐limited settings. There is an increasing interest in using CXR as a cheap pulmonary tuberculosis triage test, especially when combined with automated AI analysis. A recent prospective study compared two commercial AI products and concluded that both products met the WHO‐recommended minimal accuracy for pulmonary tuberculosis triage tests.^65^

#### AI for quantification

Automatic quantification of disease is another area for AI applications in thoracic imaging. Exemplary applications are automatic emphysema quantification,^66^ automatic calcium scoring,^67^ or quantification of diffuse lung diseases.^68^ These applications provide accurate and reproducible quantification results that may (e.g., in the case of automatic calcium scoring) be used for cardiovascular disease risk prediction.^69^

### AI in thoracic imaging: where do we stand?

What can AI do and what can it not do? An often heard rule of thumb for what AI can do, introduced by Andrew Ng, is: ‘If a typical person can do a mental task with less than one second of thought, we can probably automate it using AI either now or in the near future’ (https://hbr.org/2016/11/what-artificial-intelligence-can-and-cant-do-right-now).^70^

Thus, specific tasks such as localization and classification of lesions, segmentation of organs and quantification of disease extent can currently be accurately performed by AI solutions. Often, these AI applications target repetitive, tedious and routine tasks for radiologists. At present, most AI products are approved for clinical use as an aid to radiologists, but the first products that perform autonomous assessment of images have entered the market.^65^, ^71^ In thoracic imaging, a commercial product that can automatically and autonomously evaluate a chest x‐ray study has recently obtained regulatory approval; the product automatically generates a report if the device is confident that the study has no actionable radiological findings.

The release of large public datasets has been crucial for the development and commercialization of AI products for thoracic imaging in the last decade. Large imaging datasets combined with high‐quality annotations can be used to develop AI products that reach performance on par or surpassing physicians for specific tasks. This has resulted in the large set of commercially available AI applications that are entering clinical care. Therefore, the availability of more data combined with high‐quality annotations will be crucially important to continue to improve AI technology and to effectively validate these products on representative multi‐centre datasets. Federated learning, a novel technique in which AI systems are trained using multiple decentralized data sources while maintaining data anonymity, may prove to be an effective technique that removes the current barriers for data sharing.^72^

Important challenges for the adoption of AI technology are integration into healthcare IT systems, current clinical workflows and reimbursement. Integration of AI systems into existing hospital systems such as the picture archiving and communications system (PACS) is one of the obstacles to widespread use of AI in hospitals. Suboptimal integration hinders efficient workflow integration and hinders possible reductions in reporting times. As radiologists are currently experiencing high workloads, extra delays resulting from AI interfacing with PACS or calling up studies are undesirable. Second, with a few exceptions, there is no reimbursement for the use of AI software. Therefore, the costs for the AI software need to be covered by the hospital, which is problematic when direct cost savings are unclear.

## X‐RAY VELOCIMETRY

X‐ray velocimetry (XV technology) integrates x‐ray (fluoroscopic) imaging with unique image processing to produce quantitative four‐dimensional (4D) measurement of lung tissue motion, and accurate calculations of lung ventilation. In brief, the fundamental measurement principles of motion used in XV technology are derived from a limited number of cinefluoroscopic projection views to yield 4D measurement of tissue motion to derive physiologically meaningful measures of ventilation. Literature outlining the development and validation of the method of measurement and analysis that comprise XV technology are multidisciplinary in nature, highlighting the innovation intersection between clinical research, engineering, physics, physiology and biomechanics. The studies essential in validating the translational approach underscore the respiratory physiological principles and clinical application of the XV lung ventilation analysis. XV has been utilized for well over a decade as a research tool and is now seeing growing use in clinical practice.

### Technical development of XV technology

The XV technology was originally developed in the mid‐2000s from innovations in the fields of experimental fluid mechanics, image processing, x‐ray physics^73^, ^74^, ^75^, ^76^ and in vivo x‐ray imaging,^77^, ^78^ designed specifically for imaging of ventilation,^79^, ^80^, ^81^, ^82^, ^83^ airway surfaces,^84^, ^85^, ^86^ mucociliary transport^87^, ^88^, ^89^ and blood flow.^90^, ^91^ Significant technical advancements that led to the derivation of XV technology were: (1) the ability to measure motion in three spatial dimensions using X‐rays acquired at a single projection angle^92^; (2) the ability to reconstruct, using multiple x‐ray projections, a three‐dimensional (3D) motion field without the need to reconstruct a 3D image of the structure^93^, ^94^ and (3) the ability to accurately and reliably calculate regional ventilation data from a 3D motion field.^95^

#### Origins in 3D flow measurement of opaque fluids

**FIGURE 4:**
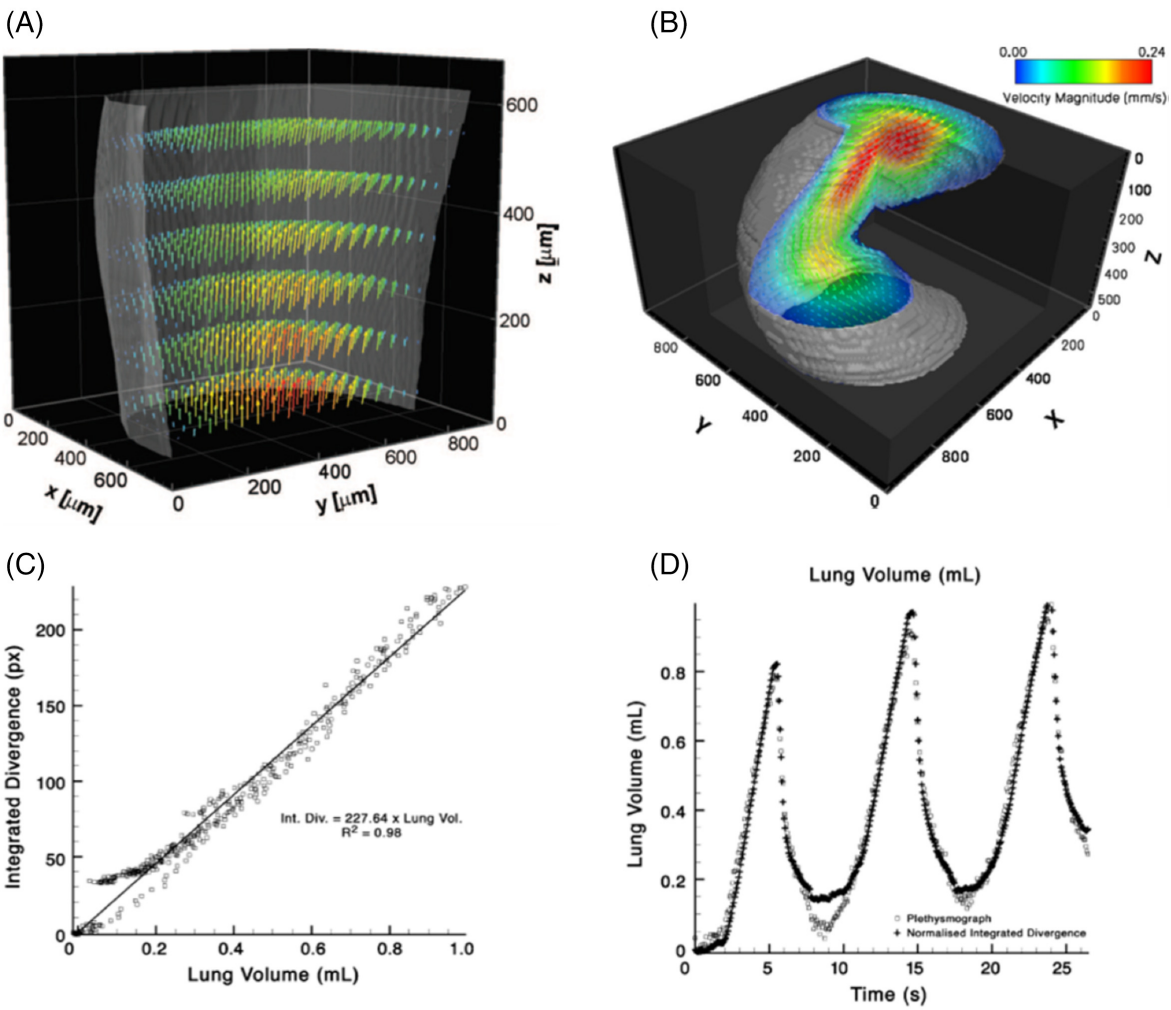
Preclinical experiments validating the accuracy and validity of lung volume measures using XV technology, with (A) and (B) showing bench‐top measurements of fluid flow validated against computer modelling; and (C) and (D) showing in vivo measurements of ventilation in rabbit lungs validated against plethysmography. (A) Reconstructed 3D blood velocity flow fields measured using XV. For clarity only half the sample is plotted, with reduced vector resolution in all dimensions. Vector colours represent velocity magnitude and are validated against computational models of the flow field. (B) CT XV reconstruction of flow field through helical geometry. A section of the result has been rendered as transparent for visualization of the flow. The results indicate the ability of CT XV to simultaneously measure the 3D structure and velocity of flow through complex geometries. (C) In vivo measurements of ventilation in rabbit lungs with validation of integrated divergence (volume) measurements from XV technology against volume measures from plethysmography. A scatter plot shows strong correlation between two quantities. (D) Time series of lung volume co‐plotted with divergence demonstrated a direct link between divergence and tissue expansion

The first breakthrough component of XV was a method to derive 3D motion measurements from two‐dimensional (2D) image sequences modified from particle image velocimetry.^92^ Investigators found that synchrotron‐based x‐ray imaging enabled accurate velocity measurement of particle‐laden, opaque fluids in regular flow, by showing that intra‐image cross‐correlation peaks from cinematic 2D x‐ray image sequence of a 3D flow represents a probability density function of velocities within the measurement volume. Furthermore, analysis of the cross‐correlation peaks facilitates accurate reconstruction of the 3D velocity field of the flow (Figure 4A). 3D velocity reconstruction from only a single 2D projection image sequence represented a significant breakthrough, and it was subsequently shown that this new methodology could be transferred from the synchrotron to smaller laboratory x‐ray systems^96^, ^97^, ^98^ and other imaging modalities, including x‐ray speckle imaging,^99^ microscopy,^100^, ^101^ holography^102^ and 2D projection images.^103^

The 3D flow measurement was extended to include the full 3D, time varying motion field reconstructed from particle image velocimetry cross‐correlation peaks derived from image sequences obtained at multiple projection views. Referred to as CT x‐ray velocimetry the method is fundamentally similar to what is now known as XV technology. This approach was used to measure blood flow through an optically opaque arterial model, with x‐ray images from multiple projection angles. Minimizing the error between the measured correlation functions and the analytically modelled correlation functions for each imaged section, facilitates 3D reconstruction of the velocity field (Figure 4B). Dubsky et al. detailed accuracy via analysis through generated image sequences containing features that undergo a mathematically calculable yet complex motion, which showed that the average error for all velocity reconstructions was less than 2% of the maximum velocity of the known input motion field.^94^

#### Regional ventilation calculation from measured motion fields

A significant milestone in XV technology is the ability to accurately and reliably calculate regional ventilation from the 3D velocity measurements based on the principle that the ventilation of the local tissue is approximated by the local tissue expansion.^95^ The ventilation assumption is predicated on the conservation of airflow entering and leaving the lungs, with the volume of transported air being closely described by the volumetric expansion of the lung tissue. Measured tissue velocities in the 3D vector field have three components (X, Y and Z) and therefore nine spatial partial derivatives. The expansion (mathematically described as the divergence, or the Jacobian) is calculated from these spatial derivatives and is a scalar value that describes the local tissue expansion and contraction at the corresponding point, demonstrated to closely approximate the specific ventilation field.

Reinhardt and co‐authors earlier presented an almost identical concept of using spatial derivatives of the motion field.^104^, ^105^ In this work, multiple volumetric CT images captured at different breath phases were used to produce a 3D motion field and the spatial derivatives are referred to as the Jacobian. Reinhardt et al.^104^ compared the Jacobian to additional measurements of regional specific ventilation obtained using Xe‐CT imaging. The Jacobian has subsequently been used in other inspiratory‐expiratory studies,^106^, ^107^ and can also be used with other technologies that involve the acquisition and comparison of multiple x‐ray CT images through the breath, in particular four‐dimensional computed tomography.^108^, ^109^, ^110^, ^111^, ^112^, ^113^

The accuracy of the divergence calculation depends strongly on both the accuracy of the raw measurements and the method with which the spatial gradients are calculated. In the case of XV technology, the gradient calculation method developed by Fouras and Soria dramatically reduces such errors from derivatives that are susceptible to increased random error due to noise (measurement error), without a proportionate loss of spatial resolution. Divergence has been validated as equivalent to ventilation. Fouras et al.^95^ used preclinical animal models to demonstrate a very strong correlation with independent plethysmographic measurements of lung volume throughout the breath (*R* ^2^ = 0.98) (Figure 4C,D).^95^

### XV technology in preclinical studies

Since its development, XV technology and its components have been applied to a large number of animal studies to measure regional lung ventilation, and to provide researchers and clinicians with useful insights into lung function and disease processes.

#### XV to quantify regional ventilation in bleomycin model of lung disease

The first reported use of XV technology to assess regional lung ventilation in animal models examined changes following exposure to bleomycin, a well characterized experimental disease model that results in pathological changes to lung structure and function.^95^ Lung tissue motion derived from x‐ray cinefluoroscopic sequences of the breathing lung was obtained in control and bleomycin‐treated mice (36‐h and 6 days after exposure) that were verified to have different pulmonary function using plethysmography‐derived lung compliance and tidal volume measurements. Moreover, histological images of control and bleomycin‐treated lungs corresponded with healthy and localized regions of pathology, respectively.

The normalized XV divergence values showed that, at 36‐h post treatment, in bleomycin‐treated mice 14% of lung regions displayed expansion twice the control average compared with less than 5% for control lungs. Furthermore, at 6 days post treatment, 47% of treated lung regions in the bleomycin group showed differences twice the control average, compared with less than 4% for saline‐treated mice. Physiologically relevant acute changes in ventilation distribution related to disease pathogenesis were evident using XV technology well before detectability by plethysmography or CT.

#### XV to study CF‐like lung disease model

XV technology is a valuable tool for the assessment of lung diseases such as CF, to investigate the functional capacity of lung tissue in healthy and transgenic β‐ENaC mice that develop a spontaneous CF‐like lung disease with airway mucus obstruction and chronic airway inflammation.^114^ Specifically, the authors used the regional expansion (volume change) to derive time‐resolved airflow measurements overlaid onto a segmented airway tree structure. Regional filling defects were identified as an area of reduced expansion and increased airflow time constant corresponding to a histological section showing mucus blockage in the bronchial tree that feeds the respective lung lobe. The anatomical association validates the ability of the regional lung ventilation map to assist in the assessment of lung health.

#### XV technology to quantify changes in an ARDS model

Using XV technology, Kim et al.^115^ investigated the contribution of mechanical ventilation in increasing the anatomic dead space in mice. Increased dead space is an important prognostic marker in early acute respiratory distress syndrome (ARDS) that correlates with mortality. The authors used XV to generate detailed 3D tissue expansion maps that allowed precise measurements of lung tidal volumes. As with previous studies, Kim et al.^115^ estimated the relative contribution of airway volumes to the total tidal volume. They showed that the airway volumes increased over time with exposure to mechanical ventilation without a concomitant increase in tidal volume. The findings suggest that anatomic dead space increases progressively with exposure to positive pressure ventilation and may represent a pathological process. XV technology allows in vivo derivation of airway volume measurements in mice, which has not been possible to date.

### Clinical use of XV technology

XV is a software only technology and requires no special equipment for image acquisition. The technology uses C‐arm fluoroscopy, a standard imaging technology found in hospitals and many outpatient radiology facilities around the world. As such, images used to generate XV ventilation reports are captured with existing conventional C‐arm fluoroscopic systems which are compatible with the XV protocol.The first clinical validation of XV technology was performed to quantify lung function in a cohort of patients undergoing radiation therapy for various thoracic cancers (excluding lung cancers).^116^, ^117^ In this study regional lung ventilation was quantified and compared to the current gold‐standard diagnostics of spirometry and CT. Specifically, changes in lung function were assessed at 4 and 12 months after radiotherapy. For each participant, fluoroscopic images of lungs during spontaneous breathing were obtained at five distinct angles across the chest (anterior–posterior, ±36° and ±72°). Image sequences were acquired to capture at least one complete, continuous breath (~4–6 s) at each of the five angles. Imaging was performed with the patient in a supine position during tidal breathing. Automatic Exposure Control setting of the detector on the x‐ray system was active to ensure the captured images were acquired with the highest level of contrast. Whenever possible, images covered the entire lungs in the field of view. Standard clinical data (including CT studies and spirometry) were collected from each patient.

**FIGURE 5:**
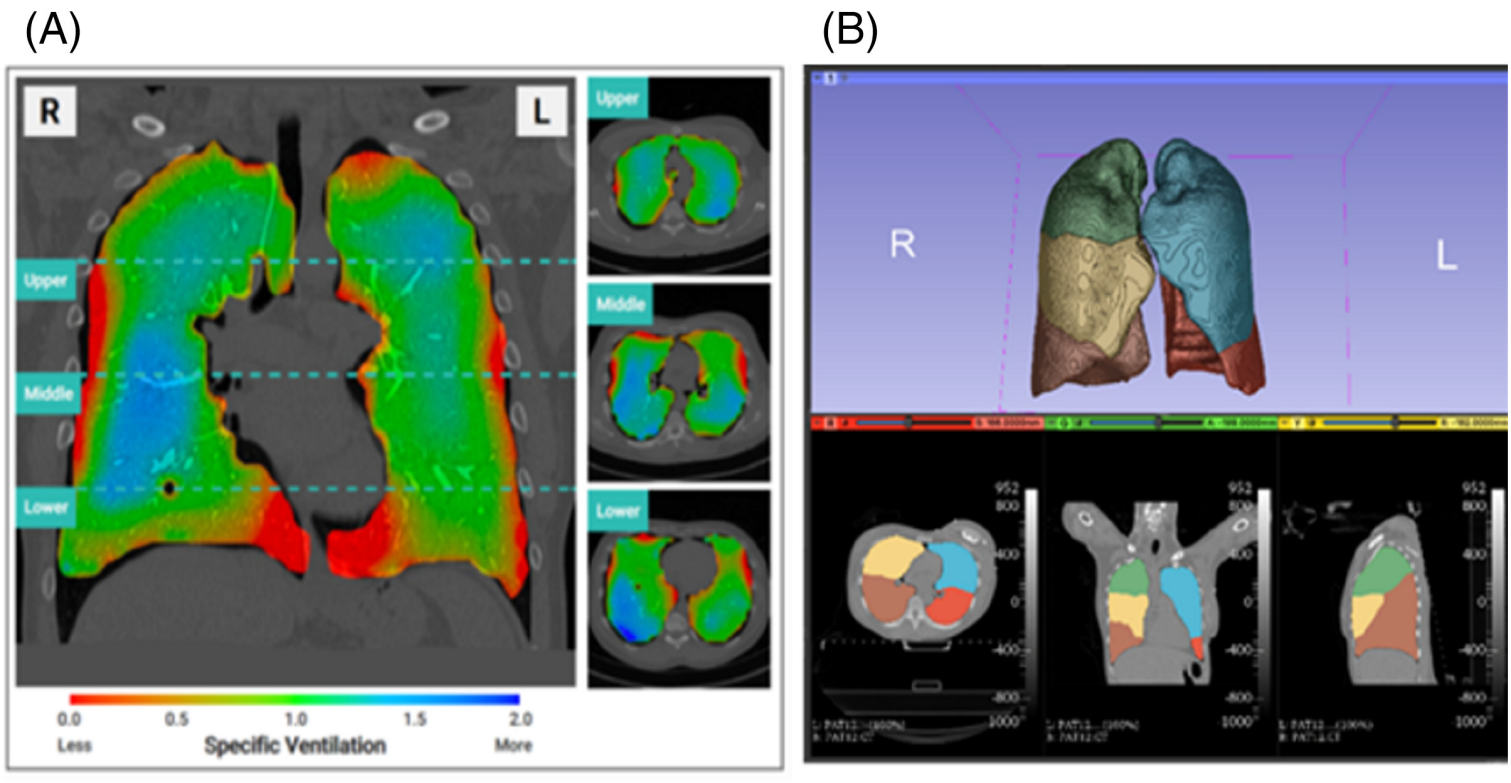
(A) Distribution of regional lung ventilation during XV scanning is shown using a colour scale where red represents underventilation, green represents average ventilation and blue represents hyperventilation relative to the mean regional lung volume expansion. The visualization maps show a mid‐coronal slice and axial slices from the upper, middle and lower zones at peak inspiration. (B) Lobe‐wise XV analysis performed using an automated anatomy‐based segmentation

The output analysis includes layers of 4D display of the volume distribution and the individual frequency distribution. Coloured maps demonstrating regional ventilation of the lungs at peak tidal inspiration were generated for each patient (Figure 5A). Additional analysis was performed to include lobar segmentation of the volume distribution (Figure 5B).

Analysis revealed correlations between XV‐derived ventilation data and CT and spirometry.^117^ Specifically, ventilation heterogeneity (a marker of how uniform or non‐uniform airflow in the lungs is) and ventilation defect percentage (which quantifies lung tissue with relatively low specific ventilation) were correlated with forced expiratory volume in 1 s (FEV_1_) and FEV_1_/forced vital capacity (FVC).^117^ More importantly, the results highlighted the advantage of the XV technology over standard tests by providing regional quantification of ventilation.^116^

When some parts of the lungs are affected by disease, healthy regions may overcompensate, masking the disease from the global functional measures. Several patients in the study with normal FEV_1_ and FEV_1_/FVC values at baseline and at 4 months after radiotherapy showed decline in lung function at 12 months. It was expected that these patients may have had a reduction in lung function as a result of radiotherapy. However, XV was demonstrated to be more sensitive in measuring alterations in regional lung function over time, with the ability to detect changes at the 4‐month timepoint.

These results indicate XV technology has a great potential to detect subtle changes in lung function earlier than available from standard pulmonary function tests. The results of this study demonstrated that not only is XV technology consistent with the standard tests, but in many cases, it offers a superior richness and sensitivity, enabling detection of even subtle functional losses well before lung structure is irreversibly affected by disease, meaning that treatment may be applied early.^117^

Furthermore, compared to standard CT protocols (not being ultra‐low‐dose, as described above), XV imaging requires a significantly lower radiation dose to the patient (as approved by the FDA, XV technology delivers 0.2–0.5 mSv, compared to a standard thoracic CT dose of 3–6 mSv, taking note that XV provides functional information, while CT provides primarily structural information) and as such can be repeated over a short time interval which is ideal for monitoring disease progression/response to treatment.

In addition, assessment of treatment outcomes using global measures is hindered, making it a major challenge for development of novel biologics or delivery methods. Furthermore, as XV imaging is performed during tidal breathing, it is less dependent on the patient's compliance compared to standard imaging and spirometry that require breath‐hold or other respiratory manoeuvres. This means that XV imaging can be offered to severely ill patients who are unable to perform breathing manoeuvres or young children who may not be able to comply with technical instructions.

This study also validated the repeatability of the measurements by comparing results (1) obtained for multiple sets of image sequences on the same day and (2) those obtained on two different days, approximately 2 weeks apart (before the initiation of radiotherapy).^118^ In the first instance, the comparison allows to gauge natural breath‐to‐breath variations and accuracy of the technique. Comparison between scans acquired on two separate days is useful in determining the repeatability of the measurements.

Both analyses demonstrated that the XV can deliver outputs with high repeatability and reproducibility. Overall, the performance, reproducibility and safety of the XV technology make it ideal for clinical and research use and future studies should determine its utility across different lung conditions.

## DISCUSSION

In this review, a selection of innovations in chest imaging have been introduced and elucidated. ULD CT techniques, with radiation dose getting close to that of a chest x‐ray examination, combined with novel reconstruction methods, have great promise for use in CT lung cancer screening. While studies so far mainly focused on image quality and lung nodule detection rate, more evidence is needed on the impact on lung nodule measurement and categorization, prior to potential implementation in screening settings. The introduction of PCD technology heralds a new era in CT imaging with much promise for chest imaging, such as high spatial resolution/low noise imaging of interstitial lung disease, and improved spectral imaging, available in each CT acquisition.

Radiomics is a fast‐evolving technology that has not yet translated to the clinics as much as was expected. It has the potential to support treatment selection while it may predict oncological outcomes such as response rate, progression free survival and overall survival. Further work needs to be done to accelerate radiomics translation, in areas such as image standardization, availability of large datasets and finally, explainable models that can gain the trust of the end user.

Deep learning technology has revolutionized the field of AI and has become the methodology of choice in medical image interpretation. A new wave of certified AI products is becoming available for clinical use and respiratory physicians will start to see radiological reports created with the help of AI. In the next 5 years, deep learning technology will evolve and AI solutions in thoracic imaging will become more mature. More and more data will need to be made available, either publicly in an anonymous dataset or via federated learning approaches. As a result, the availability and performance of AI products in thoracic imaging will evolve. Prospective multi‐centre validation studies will be performed to estimate the clinical impact of AI solutions on patient outcome and the results of these studies will be crucial for the adoption and reimbursement of AI software.

XV technology provides a platform for the 4D measurement of lung tissue motion, and subsequent calculation of lung ventilation. XV has been successfully validated through a range of preclinical and clinical studies, providing insight into respiratory diseases and treatments, as well as complex physiological processes. Importantly, these studies have verified the accuracy of the XV technology, quantified the measurement uncertainties, demonstrated repeatability and validated the technology against existing gold standard methods.

The described innovations in chest imaging also have important interactions, for example, AI solutions for automated lung nodule detection may help the introduction of ULD CT in the clinical realm, and optimized spectral evaluation in PCD CT may have added value in radiomics analyses of lung tumours. This necessitates increasing multidisciplinary collaboration between the clinical, biomedical and technical realms, to together bring the potential of new techniques and indications in chest imaging further and into the clinic.

## CONFLICTS OF INTEREST

Rozemarijn Vliegenthart is supported by an institutional research grant from Siemens Healthineers. Colin Jacobs is supported by an institutional research grant from MeVis Medical Solutions and his institution receives royalties from MeVis Medical Solutions for the development of Veolity, a reading platform for lung cancer screening. Nikolaos Papanikolaou is the owner of MRIcons LTD. Andreas Fouras is a founder and CEO of 4DMedical, a global medical technology company.
